# Data‐informed Stepped Care (DiSC) to improve adolescent and young adult HIV care outcomes in Kenya: a cluster randomized trial

**DOI:** 10.1002/jia2.26501

**Published:** 2025-07-07

**Authors:** Pamela Kohler, Wenwen Jiang, Jacinta Badia, James Kibugi, Jessica Dyer, Julie Kadima, Dorothy Oketch, Kristin Beima‐Sofie, Sarah Hicks, Barbra A. Richardson, Irene Inwani, Seema K. Shah, Kawango Agot, Grace John‐Stewart

**Affiliations:** ^1^ University of Washington Seattle Washington USA; ^2^ IMPACT Research and Development Organization Kisumu Kenya; ^3^ Kenyatta National Hospital Nairobi Kenya; ^4^ Northwestern University Feinberg School of Medicine Chicago Illinois USA; ^5^ Lurie Children's Hospital Chicago Illinois USA

**Keywords:** adolescents and young adults, ART adherence, differentiated service delivery, retention, stepped care, viral suppression

## Abstract

**Introduction:**

Systematic use of data‐driven tools to allocate care services based on needs, including differentiated care for stable individuals and intensive care for those with higher risk, may improve retention and viral suppression in adolescents and young adults living with HIV (AYLHIV).

**Methods:**

This cluster randomised trial in western Kenya tested a data‐informed stepped care intervention that assigned AYLHIV to four intensities of care according to need. AYLHIV at 12 intervention facilities underwent step assignment at each visit; those at lowest risk were offered differentiated models of service delivery (DSD), and those with risk factors more intensive services. AYLHIV at control sites received standard care. AYLHIV were followed for 12 months. Clinical and viral load data were abstracted from medical records. The primary outcome was the proportion of missed visits (defined as > 30 days late for scheduled visit). Secondary outcomes included loss to follow‐up, viral non‐suppression and assignment to DSD (multi‐month refills or pharmacy fast‐track visits). Mixed effects regression was clustered by individual and facility and adjusted for outcomes during the pre‐enrolment period and baseline variables that differed by arm.

**Results:**

Between April and July 2022, 1911 AYLHIV ages 10–24 were enrolled (control: 1016, intervention: 895, 1708.8 person‐years). Median age was 17, and 1512 (79.5%) were in school. Characteristics were balanced by arm, except for a higher proportion coming to the clinic alone in control arm (68.5% vs. 61.1%, *p* = 0.04). At intervention facilities, using the DiSC tool, 574 (64.6%) AYLHIV were assigned to DSD, 122 (13.7%) to standard care, 100 (11.3%) to mental health and retention counselling, and 92 (10.4%) to intensive case management. Missed visits were 8.5% in intervention versus 8.3% in control (adjusted risk ratio [aRR]: 1.04, 95% CI: 0.89−1.20); viral non‐suppression (7.7% vs. 9.7%, aRR 0.79 95% CI: 0.54−1.16) and antiretroviral therapy adherence (92.8% vs. 94.6%, aRR 0.98 95% CI: 0.94−1.02) were similar between arms. AYLHIV in the intervention arm received more fast‐track visits (aRR 1.21, 95% CI: 1.01−1.46). Intervention facilities experienced fewer scheduled appointments compared to control (aRR: 0.95, 95% CI: 0.91−0.98, *p* = 0.004).

**Conclusions:**

Overall, missed visits and non‐suppression were infrequent (< 10%) and did not decrease with the DiSC intervention. The DiSC intervention resulted in increased assignment to differentiated services without increasing missed visits or viral non‐suppression.

## 1 INTRODUCTION

There are an estimated 3.7 million adolescents and young people (ages 10–24) living with HIV worldwide, of whom 82% live in sub‐Saharan Africa [1]. Globally, adolescents and young adults living with HIV (AYLHIV) experience disproportionately poor care outcomes—including viral non‐suppression and lower retention in care—compared to other age groups [2, 3]. In Kenya, 56% of 15‐ to 24‐year olds living with HIV are virally suppressed, compared to 71% of those ages 25–49 [4].

The World Health Organization (WHO) recommends differentiated models of service delivery (DSD) for HIV treatment as a client‐centred approach of simplifying and adapting HIV services across the care cascade to meet population needs and reduce the burden on the health system [5, 6]. DSD models may be group‐focused (managed by healthcare workers [HCWs] or clients) or individual‐focused (based at facilities or not) and may include multi‐month refills, pharmacy fast‐track visits and/or community‐based antiretroviral therapy (ART) delivery [7]. However, due to concerns over poor care outcomes, some countries have classified adolescents as “unstable” or otherwise ineligible for DSD [8, 9].

The adolescent and young adulthood life stages are a transitional time of increasing independence in seeking care and management of health [7, 8]. Those enrolled in school, particularly boarding schools which are common in Kenya and result in youth being away from home for most of the year, face additional challenges in navigating treatment schedules that may not align with school breaks [9]. Despite being not widely available to AYLHIV, differentiated approaches may better meet the needs of this population and free up HCW time to provide more intensive care to youth needing additional support.

Two different evidence‐based interventions may offer a data‐driven approach to safely tailor AYLHIV care to population needs: clinical prediction tools and stepped care services. Clinical prediction tools use cohort or programme data to classify individuals into high‐ and low‐risk categories, thereby identifying those in need of intervention prior to the occurrence of poor outcomes [10, 11, 12, 13]. Stepped care involves a process in which HCWs assign clients to different intervention steps according to their level of need [14]. The model has been used in mental healthcare to align client services with effective and appropriate interventions, starting with low‐intensity treatments and systematically progressing to higher levels of care based on client monitoring [15, 16, 17]. Thus, a prediction tool for loss to follow‐up could enable HCWs to identify youth at risk for missed visits and be used to assign youth into appropriate steps of service delivery—including less frequent or differentiated services, according to need.

This study aims to evaluate a data‐driven, stepped care intervention assigning AYLHIV in Kenya to more‐ or less‐intensive service steps.

## 2 METHODS

### 2.1 Study design

This was an unblinded 2‐arm cluster randomised clinical trial of a multicomponent stepped care intervention conducted at 24 HIV care and treatment facilities located in western Kenya. The trial protocol was previously published [18] and registered at clinicaltrials.gov (NCT05007717). The cluster design was used because the intervention was adopted at the health facility level and not the individual client level. Clusters were defined as health facilities that provide HIV care and treatment to AYLHIV. Intervention facilities implemented the clinical prediction tool plus stepped care model, while control sites continued their current usual care.

### 2.2 Population and setting

Eligible clinics were located in Kisumu, HomaBay or Migori County, Kenya; had at least 100 AYLHIV enrolled in care, a functional electronic medical record (EMR) system and interest in participating. Adolescent and young adult clients were recruited from study sites; eligible participants were age 10–24 years old, enrolled in HIV care and aware of their HIV diagnosis.

### 2.3 Randomization

Participating clinics were randomised in a 1:1 ratio using computer‐generated random numbers to either intervention or control, using restricted randomization to balance county and facility size between arms. The timing of randomization was prior to the study start. Randomization was done at the University of Washington by a biostatistician not involved in study procedures. A list of clinics with their allocation arms was sent to the study team. The intervention was administered at the clinic level; it was not possible to blind participating clinics or study team members.

### 2.4 Intervention

The Data‐informed Stepped Care (DiSC) tool was a 10‐item clinical risk assessment tool (Supporting information) that assigned a score based on risk for loss to follow‐up at each clinic visit. Based on risk score, AYLHIV were assigned to different levels of care, or “steps” (Table 1). Services ranged from a relative position of increased AYLHIV autonomy to more intensive service provision for those at the highest risk. Low‐risk AYLHIV were assigned to Step 1 if they were on ART for 12 months, had no opportunistic infection in the last 6 months and were virally suppressed at the last measure. Participants were eligible for Step 2 (standard of care) based on ineligibility for Step 1, duration enrolled in care < 6 months, client preference, or pregnant or breastfeeding status. Step 3 was assigned to those reporting depression as evaluated by standardised Patient Health Questionnaire‐2 (PHQ‐2) screening or having missed more than one clinic visit in the last 6 months. Step 4 clients were those with current unsuppressed viral loads (VLs). Services assigned to each step included: Step 1: differentiated services (fast‐track pharmacy visits and longer visit intervals); Step 2: job tools for standard of care, support groups, standard visit intervals; Step 3: motivational interviewing and/or psychosocial counselling; and Step 4: adherence counselling, case management, transportation support and/or home visits. Psychosocial counselling sessions were brief, flexible, delivered by routine HIV care providers or adherence counsellors over five sessions, and adapted from problem‐solving and cognitive‐based therapy interventions [19, 20].

**Table 1:** Overview of step eligibility and services

Step	Eligibility	Services offered
1	On antiretroviral therapy × 12 months [AND] No opportunistic infections (OIs) in last 6 months [AND] Viral suppression at last measure	Differentiated services Longer visit intervals (when)^a^ Pharmacy fast‐track (where/who)
2	Does not meet criteria for Step 1 [OR] Client preference for standard care [OR] Pregnant/breastfeeding	Standard care Job tools: OI, pregnancy, newly enrolled Support groups (what) Standard visit intervals (when)
3	Depression (Patient Health Questionnaire‐2 [PHQ‐2] ≥ 3) [OR] Missed one or more visits in last 6 months	Individual counselling Motivational interviewing (what) Psychosocial counselling (what)
4	Unsuppressed viral load (VL)	Intensive support services Adherence counselling (what) Case management (what) Transportation support (what) Home visits (what)

^a^ when/where/who/what refers to relevant differentiated models of service delivery (DSD) building blocks.

The intervention was integrated into routine care, with facility HCWs employed by the participating sites conducting the loss to follow‐up (LTFU) risk assessment, step assignment and delivering services. Control sites continued with standard‐of‐care approaches for adolescent clinic visits (usually 1–3 monthly visits) regardless of healthcare needs, providing additional support as needed.

### 2.5 Data collection

Medical record data from all AYLHIV with at least one clinic record within the 12‐month period prior to the first data abstraction date were included. EMR data abstraction was conducted at the facility or at the office of the technical partner that supports the EMR system, at baseline and every 4 months throughout the study. Routine programmatic HIV VL testing data (done 6 months after ART initiation, then annually) were abstracted from the national laboratory database of the National AIDS & STI Control Programme and/or the EMR, if available. Questionnaires were conducted to collect data on client anthropometric measures, socio‐demographics, healthcare use, sexual behaviour, depression (using Patient Health Questionnaire 9 [PHQ‐9]) [21], anxiety (using General Anxiety Disorder‐7 [GAD‐7]) [22], drug use (using WHO Alcohol, Smoking and Substance Involvement Screening Test for Young people [ASSIST‐Y]) [23], social support (using Multidimensional Scale of Perceived Social Support [MSPSS] 12‐item scale) [24], resilience (using Connor‐Davidson Resilience Scale‐2 [CDRS‐2]) [25], adherence self‐efficacy (using Adherence Self‐Efficacy Scale [ASES]) [26], social stigma (using Youth HIV Brief Stigma Scale [YHBSS]) [27], HIV disclosure, HIV testing history, CD4 count and clinical history. Transfer‐out status and treatment discontinuations were actively reviewed. Surveys were administered at enrolment and every 6 months thereafter, either in‐person or over the phone by a trained study staff.

### 2.6 Study outcomes

The primary trial outcome was missed visits, defined as a participant being > 30 days late for a scheduled visit. Secondary outcomes included loss to follow‐up, defined as a participant not seen within 30 days of a scheduled visit and not returning to care within the 12‐month study interval; viral non‐suppression, defined as HIV VL > 1000 copies/ml; receipt of differentiated services (defined either as multi‐month refills with long inter‐visit intervals or “fast‐track” pharmacy visits for expedited ART pick‐up without a full clinical evaluation); and ART adherence (defined as the proportion of days with ART coverage between the most recent ART refill and the next scheduled refill date (> 80% indicating good adherence).

### 2.7 Statistical analyses

The trial sample size was calculated for 80% power to detect a 14−17% increase in retention, assuming alpha = 5% and a coefficient of variation of 0.1−0.15. Missed visits were compared between study arms using mixed effects Poisson regression, clustered by individual and facility. Time to loss to follow‐up was compared between arms using survival curves and Cox proportional hazards regression, with censoring of deaths or transfers. Viral non‐suppression, receipt of DSD and ART adherence were each compared between study arms using mixed effects Poisson regression clustered by individual and facility. Proportions of participants ever missed a visit, ever received DSD or always good adherence were each compared between study arms using mixed effects Poisson regression clustered by facility. All analyses were intent‐to‐treat, using robust standard errors, adjusted for baseline level of corresponding outcomes during a 6‐month pre‐enrolment period and variables that differed by arm at study enrolment. All analyses were conducted using RStudio Version 1.2.5042 (RStudio, Inc).

### 2.8 Ethical considerations

Ethical approval was obtained from the University of Washington (STUDY00011096), Maseno University Ethical Review Committee (MUERC/00917/20) and the Kenya National Commission for Science, Technology and Innovation (NACOSTI 444824). Study staff obtained written consent/assent before assessing participant eligibility. If AYLHIV ages < 18 were brought to the clinic by a caregiver, the caregiver provided written informed consent and the AYLHIV assented to participate. AYLHIV ages < 18 who met the definition of emancipated minor (pregnant/has child, married/partnered, financially or otherwise independent of parents) or mature minor (age 15, living apart from parents and managing own finances) under Kenyan law were able to provide written consent as adults. A waiver of parental permission was obtained for AYLHIV ages 15–17 years who did not meet emancipated or mature minor criteria and whose caregivers were not present. Enrolment for all AYLHIV ages < 15 years of age required caregiver consent. Severe adverse events were monitored and reported to the trial Data Safety Monitoring Board and study monitors in real time.

## 3 RESULTS

**Figure 1:**
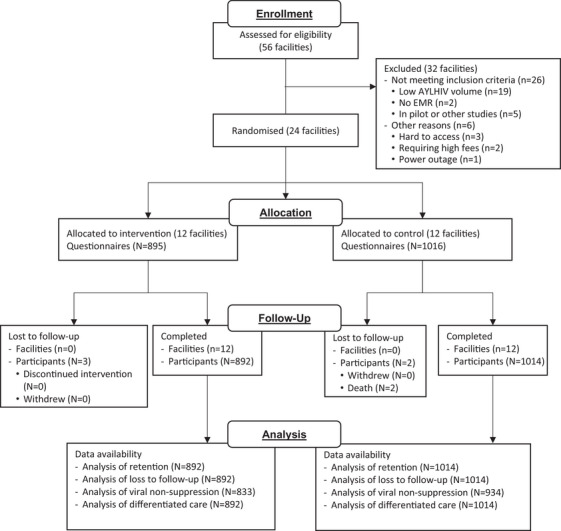
**Consort diagram**. Abbreviation: AYLHIV, young adults living with HIV.

Between 19 April 2022 and 19 July 2022, 1974 AYLHIV were screened for eligibility at 24 study facilities. Of those screened, 1967 AYLHIV (99.6%) were found to be eligible for the study and 1911 (97.2%) were enrolled, with 895 AYLHIV enrolled from the 12 intervention facilities randomised to receive DiSC stepped care, and 1016 AYLHIV enrolled from the 12 control facilities randomised to receive standard of care (Figure 1). The number of participants enrolled per facility was between 51 and 113. Enrolled AYLHIV were followed until 19 September 2023 and contributed 1708.8 person‐years of follow‐up. Five AYLHIV died during study follow‐up (three in the intervention arm and two in the control arm). Among the intervention arm, 888 AYLHIV actually received their allocated intervention (99.2%).

### 3.1 Baseline participant characteristics

Median age was 17 years (interquartile range [IQR] 14–19) and 1102 (57.9%) were girls and young women (Table 2). Most AYLHIV were currently in school (79.5%) and most reported having at least one parent alive (83.4%). The majority (98.2%) of AYLHIV had disclosed their HIV status to other people. Few AYLHIV reported mental health issues, with 8.3% having at least mild depression (PHQ‐9 score ≥ 5), 3.7% at least mild anxiety (GAD‐7 score ≥ 5), and 7.7% reporting any use of alcohol or drugs. Almost all AYLHIV reported high social support (95.6%, MSPSS score > 3) and high adherence self‐efficacy (98.6%, ASES score ≥ 5), and 48.1% were part of a peer support group. Usage of the DSD model at baseline was reported by 35.1% of AYLHIV, and 57.9% reported having an unmet service need. Regarding ART use, 38.3% were on a dolutegravir‐based regimen, 21.7% were on an efavirenz‐based regimen, 28.1% were on a nevirapine‐based regimen and 8.1% were on lopinavir/ritonavir‐based regimen. Characteristics were balanced by randomization arm, with the exception of a higher proportion of AYLHIV self‐reporting always coming to the clinic by themselves in control versus intervention arms.

**Table 2:** Participant baseline characteristics

Characteristic	Control (*n* = 1016) *n (%) or median (IQR)* ^a^	Intervention (*n* = 888)^^^ *n (%) or median (IQR)*	Overall (*N* = 1904) *n (%) or median (IQR)*
Age (years)	17.0 (15.0, 19.0)	16.0 (14.0, 19.0)	17.0 (14.0, 19.0)
Gender			
Boys/young men	443 (43.6%)	353 (39.8%)	796 (41.9%)
Girls/young women	570 (56.2%)	532 (60.0%)	1102 (57.9%)
Currently in school	806 (79.6%)	706 (79.5%)	1512 (79.5%)
At least one parent alive	854 (84.1%)	734 (82.8%)	1588 (83.4%)
At least one parent died	570 (56.1%)	504 (56.8%)	1074 (56.4%)
Depression^b^			
None/minimal (0−4)	906 (91.3%)	789 (92.1%)	1695 (91.7%)
At least mild (5+)	86 (8.7%)	68 (7.9%)	154 (8.3%)
Anxiety^c^			
None/minimal (0−4)	967 (96.9%)	830 (95.5%)	1797 (96.3%)
At least mild (5+)	31 (3.1%)	39 (4.5%)	70 (3.7%)
Any alcohol or drug use in life^d^	79 (7.8%)	68 (7.7%)	147 (7.7%)
Social support^e^	924 (96.6%)	777 (94.5%)	1701 (95.6%)
Resilience^f^	930 (93.2%)	799 (92.1%)	1729 (92.7%)
Adherence self‐efficacy^g^	979 (98.9%)	856 (98.3%)	1835 (98.6%)
Stigma^h^	351 (37.1%)	266 (33.0%)	617 (35.2%)
Part of a peer support group	499 (49.7%)	404 (46.2%)	903 (48.1%)
Violence in the last 6 months	54 (5.3%)	62 (7.0%)	116 (6.1%)
Transport to clinic ≥ 1 hour	479 (47.9%)	351 (40.4%)	830 (44.5%)
Wait time in clinic ≥ 1 hour	97 (9.6%)	59 (6.7%)	156 (8.2%)
Use at least one differentiated service delivery (DSD) model^i^	334 (33.0%)	331 (37.4%)	665 (35.1%)
Report service needed but not received	580 (57.1%)	523 (58.9%)	1103 (57.9%)
Able to come for appointments without help from caregiver	794 (84.3%)	687 (79.3%)	1481 (81.9%)
Able to take medication without reminders from caregiver	892 (94.9%)	778 (90.4%)	1670 (92.7%)
Always coming to this clinic by yourself^***^	694 (68.6%)	541 (61.1%)	1235 (65.1%)
Other person in charge of healthcare decision	759 (75.0%)	684 (77.4%)	1443 (76.1%)
Know HIV status of last partner	258 (75.2%)	178 (73.3%)	436 (74.4%)
Disclosed to anyone	995 (97.9%)	874 (98.4%)	1869 (98.2%)
Antiretroviral therapy regimen			
Dolutegravir (DTG)‐based^j^	348 (35.4%)	362 (41.7%)	710 (38.3%)
Efavirenz (EFV)‐based^k^	222 (22.6%)	179 (20.6%)	401 (21.7%)
Lopinavir/ritonavir (LPV/r)‐based^l^	89 (9.1%)	61 (7.0%)	150 (8.1%)
Nevirapine (NVP)‐based^m^	281 (28.6%)	240 (27.6%)	521 (28.1%)
Other	43 (4.4%)	27 (3.1%)	70 (3.8%)

^a^ Interquartile range.

^b^ Evaluated using Patient Health Questionnaire‐9 (PHQ‐9) with a score of 0–4 indicating none/minimal depression, 5–9 mild depression, 10–14 moderate depression, ≥ 15 severe depression.

^c^ Evaluated using General Anxiety Disorder‐7 (GAD‐7) with a score of 0–4 indicating none/minimal anxiety, 5–9 mild anxiety, 10–14 moderate anxiety, ≥ 15 severe anxiety.

^d^ Using WHO Alcohol, Smoking and Substance Involvement Screening Test for Young people (ASSIST‐Y).

^e^ Evaluated using Multidimensional Scale of Perceived Social Support (MSPSS) 12‐item scale with a score > 3 indicating high social support.

^f^ Evaluated using Connor‐Davidson Resilience Scale‐2 (CDRS‐2) with a score ≥ 3 indicating high resilience.

^g^ Evaluated using Adherence Self‐Efficacy Scale (ASES) with a score ≥ 5 indicating high adherence.

^h^ Evaluated using Youth HIV Brief Stigma Scale (YHBSS) with a score ≥ 3 indicating high stigma experience.

^i^ Differentiated service delivery.

^j^ Dolutegravir.

^k^ Efavirenz.

^l^ Lopinavir/ritonavir.

^m^ Nevirapine.

^ Seven participants in the intervention arm missed the baseline survey.

*** Significance by arm estimated by generalised linear mixed model (GLM regression clustered by facility; *p*‐value < 0.05.

Among AYLHIV in the intervention arm, at baseline, 574 (64.6%) were assigned to Step 1 as stably viral suppressed; 122 (13.7%) were assigned to Step 2 as requiring standard care or declining other steps; 100 (11.3%) were assigned to Step 3 needing mental health or retention support; and 92 (10.4%) were assigned to Step 4 with unsuppressed VL.

### 3.2 Clinical outcomes

#### 3.2.1 Missed visits

A total of 1906 (99.7%) AYLHIV had at least one clinic appointment scheduled during study follow‐up, with 10,032 scheduled visits included in analyses. AYLHIV had a median of five scheduled visits (IQR 4–6). During follow‐up to 12 months, 91.6% of visits were attended within 30 days of scheduled dates. The proportion of missed visits was 8.3% in the control arm and 8.5% in the intervention arm. There was no significant difference in missed visits between arms (adjusted risk ratio [aRR]: 1.04 [0.89−1.20], *p* = 0.594). Overall, 36.8% of AYLHIV ever missed a visit (> 30 days from scheduled date), and the proportions were similar between AYLHIV enrolled from intervention facilities and AYLHIV enrolled from control facilities (Table 3).

**Table 3:** Effects of DiSC intervention on study outcomes

***Primary outcome***				
	Overall (*N* = 1906)	Control (*n* = 1014)	Intervention (*n* = 892)	Intervention versus control
**Missed visits**				
Number of scheduled visits	10,032	5248	4784	–
Proportion of missed visits/scheduled visits	839 (8.4%)	433 (8.3%)	406 (8.5%)	aRR^a^1.04 (0.89−1.20); *p* = 0.631
Proportion of AYLHIV ever missed a visit	701 (36.8%)	360 (35.5%)	341 (38.2%)	aRR^a^ 1.08 (0.92−1.25); *p* = 0.340

***Secondary outcomes***				
**LTFU**				
Proportion of AYLHIV lost to follow‐up	7.0%	7.2%	6.8%	aPR^b^ 1.00 (0.71−1.40); *p* = 0.987
Incidence rate/100py	7.84	8.00	7.67	aHR^b^ 1.00 (0.71−1.41); *p* = 0.991

	Overall (*N* = 1767)	Control (*n* = 934)	Intervention (*n* = 833)	Intervention versus control
**Viral non‐suppression**				
Number of VL assays	3523	1899	1624	–
Proportion of non‐suppression results of all VL assays	309 (8.8%)	184 (9.7%)	125 (7.7%)	aRR^c^ 0.79 (0.54−1.16); *p* = 0.237
Proportion of AYLHIV ever virally unsuppressed	219 (12.4%)	123 (13.2%)	96 (11.5%)	aRR^c^ 0.80 (0.52−1.25); *p* = 0.329

	Overall (*N* = 1904)	Control (*n* = 1013)	Intervention (*n* = 891)	Intervention versus control
**ART adherence (pill coverage > 80%)**				
Number of inter‐visit intervals with pill data recorded	9123	4741	4382	–
Proportion of inter‐visit intervals with good adherence	8551 (93.7%)	4486 (94.6%)	4065 (92.8%)	aRR^d^ 0.98 (0.94−1.02); *p* = 0.386
Proportion of AYLHIV always with good adherence	1479 (77.7%)	810 (80.0%)	669 (75.1%)	aRR^d^ 0.94 (0.85−1.05); *p* = 0.299

***Differentiated service delivery***				
	Overall (*N* = 1906)	Control (*n* = 1014)	Intervention (*n* = 892)	Intervention versus control
**Long‐interval visits**				
Number of scheduled visits	11,033	5760	5273	–
Proportion of long‐duration interval among all scheduled visits	4119 (37.3%)	2180 (37.8%)	1939 (36.8%)	aRR^e^ 1.04 (0.97−1.12); *p* = 0.276
Proportion of AYLHIV ever given long‐duration schedules	1621 (85.0%)	838 (82.6%)	783 (87.8%)	aRR^e^ 1.10 (0.90−1.35); *p* = 0.353
**Fast‐track visits**				
Number of visits with non‐missing care model	9549	4963	4586	–
Proportion of fast‐track visits of all visits	1037 (10.9%)	520 (10.5%)	517 (11.3%)	aRR^f^ 1.21 (1.01−1.46); *p* = 0.037
Proportion of AYLHIV ever having fast‐track visits	611 (32.1%)	264 (26.0%)	347 (38.9%)	aRR^f^ 1.61 (0.68−3.83); *p* = 0.278

Abbreviations: (a)HR, (adjusted) hazard ratio; (a)PR, (adjusted) prevalence risk; (a)RR, (adjusted) relative risk; ART, antiretroviral therapy; AYLHIV, adolescents and young adults living with HIV; DiSC, Data‐informed Stepped Care; LTFU, loss to follow‐up; py, person‐years; VL, viral loads.

^a^ Adjusted for attending clinic alone and *ever* having missed visits at baseline.

^b^ Adjusted for attending clinic alone; no adjustment for baseline LTFU.

^c^ Adjusted for attending clinic alone and *ever* being virally unsuppressed at baseline.

^d^ Adjusted for attending clinic alone and *always* having > 80% pill coverage at baseline.

^e^ Adjusted for attending clinic alone and *ever* being given a long duration interval visit at baseline.

^f^ Adjusted for attending clinic alone and *ever* being given fast‐track visits at baseline.

#### 3.2.2 Loss to follow‐up

**Figure 2:**
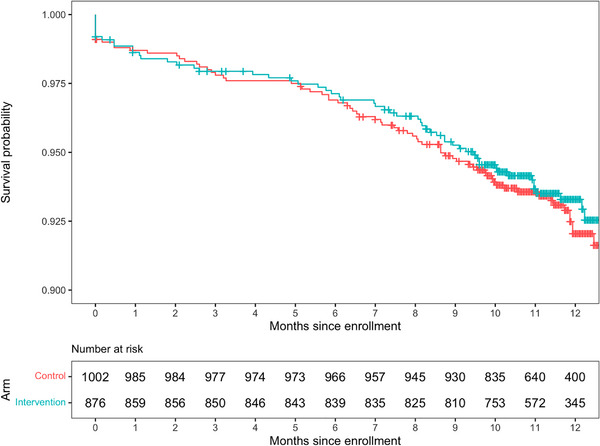
**Time to loss to follow‐up**.

Incidence of LTFU was 7.84/100 person‐years (py), with no difference between control (8.00/100py) and intervention (7.67/100py, adjusted hazard ratio [aHR]: 1.00 [0.71−1.41], *p* = 0.991) (Table 3 and Figure 2).

#### 3.2.3 Viral non‐suppression

Overall, 1767 AYLHIV had ≥ 1 VL testing result available, for a total of 3523 VL results included in analyses. AYLHIV had a median of 2 VL results (IQR 1–2). The overall proportion of VL results that were non‐suppressed was 8.8%, and 12.4% of AYLHIV were ever virally unsuppressed (Table 3). There were no differences in the frequency of non‐suppression over time between the intervention and control arm (aRR: 0.79 [0.54−1.16], *p* = 0.237). Sensitivity analyses stratified by age (10−19 and 20–24) yielded similar results.

#### 3.2.4 DSD

Among 1906 AYLHIV with clinical visit data available, a total of 11,033 visits were included in the analysis of long inter‐visit intervals (next visit scheduled for ≥ 3 months due to multi‐month dispensing). The proportion of scheduled visits with long inter‐visit interval was 37.3% and similar between arms (aRR: 1.04 [0.97−1.12], *p* = 0.276) (Table 3). For the analysis of visits with “fast‐track” care model, 10.9% of the total 9549 visits with data on care models were observed. AYLHIV in the intervention arm had a higher proportion of receiving “fast‐track” models than AYLHIV in the control arm (aRR: 1.21 [1.01−1.46], *p* = 0.037).

#### 3.2.5 Adherence

Among all clinic visits, 8551 visits (93.7%) were attended soon enough after the preceding visit that > 80% of days in the interval were covered by ART doses dispensed at the preceding visit. There were no significant differences between intervention and control arm (aRR: 0.98 [0.94−1.02], *p* = 0.386). Most (77.7%) of AYLHIV always had > 80% pill coverage during study follow‐up (Table 3). Results were similar in sensitivity analysis using 95% as the cutoff (data not shown).

#### 3.2.6 Clinic burden

At baseline, control sites averaged 34 AYLHIV visits per clinic per month, and intervention sites averaged 33 visits per clinic per month. Adjusting for 6 months pre‐enrolment at baseline, facilities implementing the DiSC tool experienced a 6% decrease in the number of visits compared to facilities in the control arm (prevalence ratio [PR]: 0.94 [0.91−0.97], *p* < 0.001).

## 4 DISCUSSION

In this cluster randomised trial, we found that a data‐driven stepped care intervention did not improve AYLHIV viral suppression or retention in HIV care. However, it was encouraging that the use of the DISC risk assessment tool was successful in assigning significantly more AYLHIV to differentiated fast‐track services than standard care, while maintaining high retention in care and viral suppression, and decreasing workforce burden.

Other studies in the region have documented the maintenance of positive HIV care outcomes and reduced costs associated with the adoption of DSD [28, 29, 30, 31, 32]. One study of DSD rollout in Uganda reported 98% retention and 91% viral suppression, with costs similar to standard of care [28]. It is hypothesised that these client‐centred models result in the reallocation of staff time and improved health system efficiency. This hypothesis is supported by our finding of significant decreases in the volume of clinic visits per site, suggesting the potential for freeing up clinic time to focus on individuals with more intensive needs while minimising workforce burden.

We found that fast‐track services increased in the intervention arm, while inter‐visit intervals were similar between arms. Fast‐track visits, where expedited pharmacy dispensing is done rather than a comprehensive evaluation, are useful to improve clinic flow and decrease wait and visit time [29], and have been shown to improve on‐time ART pick‐up [30]. In our study, it is possible that clinicians in the intervention arm may have felt more comfortable allocating ALYHIV to fast track after using the systematic DiSC stepping tool. Because of COVID‐19 and changing guidelines, inter‐visit intervals were longer in both arms than anticipated in the trial design phase, which may have contributed to the lack of difference between arms. In addition, our age range included a sizeable number of AYLHIV aged 20–24 who were already eligible for longer inter‐visit intervals, making the intervention less impactful in this group.

While some elements of DSD, specifically visit intervals aligned with school schedules, have been implemented in this setting, some providers also may feel reluctance to not see AYLHIV as frequently as monthly, with concerns over loss to follow‐up and poor adherence, and fear that they will not come back if away for too long. Interestingly, in Sierra Leone, monthly refill requirements were associated with a higher frequency of viral failure (67%) compared to longer refill durations of 2–3 months (33%) [31]. A study in South Africa of even longer refill durations found similarly high (> 90%) retention and suppression rates among those attending community‐based refills at 6 and 12 months [32]. These findings suggest that longer refill durations may be beneficial in maintaining viral suppression, particularly among young people attending boarding schools.

In this clinical trial, we did not find that the intervention improved retention (the primary outcome of the trial) or viral suppression. This may be due to generally high retention (> 90% of scheduled visits were attended) and viral suppression (> 90% of VLs assessed were suppressed) in the cohort, making it difficult to discern a small difference between trial arms despite the large sample size. However, more than a third of ALYHIV ever missed a visit and 12% of AYLHIV were unsuppressed at some point during follow‐up, suggesting continued need for programmatic improvements. Our intervention may not have had sufficient time to exert improvement in retention or viral suppression for some AYLHIV who were stepped later in the study period. Alternatively, our intervention services (mental health counselling and adherence counselling) may not have differed substantially from existing similar services in control clinics or may not have addressed residual structural issues that challenge retention and adherence. Long‐acting (LA) ART may address some of these issues, but implementation may take time and it is likely that some AYLHIV will still face challenges consistently accessing LA‐ART. Our study contributes to a body of research that suggests longer duration between visits does not negatively affect treatment outcomes.

Our study has strengths and limitations. Strengths of the study include that it was a large cluster randomised trial that combined implementation science strategies to optimise the intervention, and that it was a pragmatic intervention delivered by clinic personnel within the context of routine care. There was likely some cross‐over of elements of the intervention, as care components—with the exception of systematic mental health counselling—existed at control sites, though not using the DiSC tool for systematic assignments based on risk. The COVID‐19 pandemic similarly forced longer visit intervals across both sites, which could explain why differences found were primarily in the fast‐track pharmacy approach. Clinical outcomes within both arms were high, suggesting potential a Hawthorne effect, or improved care and support based on the knowledge that this was being observed, at control sites [33]. Expanding consent to include young people ages 15–17 seeking care independently may have improved the representativeness of the age group; however, it may also have contributed to the selection of a more stable care‐seeking population overall.

## 5 CONCLUSIONS

We found relatively high rates of retention and viral suppression in this cohort of AYLHIV which were not improved by use of the DiSC intervention in Kenya. The DiSC tool effectively identified AYLHIV doing well in care who were shifted to differentiated services, while also identifying those in need of more intensive counselling and intervention. The use of the DiSC tool improved the implementation of differentiated services among AYLHIV without compromising retention or viral suppression. Future trials in other settings, including community‐based approaches, or with additional key populations will inform further implementation and health policy.

## COMPETING INTERESTS

BAR is a member of the Data and Safety Monitoring Board for Gilead Sciences clinical trials of pre‐exposure prophylaxis. The remaining authors have no additional conflicts of interest to declare.

## AUTHORS’ CONTRIBUTIONS

PK, GJ‐S, KA, BAR, SKS and KB‐S were responsible for conceptualisation and methodology. JB, JKi, JD, JKa, DO and SH contributed to study implementation and data collection. WJ and BAR contributed to data analysis. PK wrote the original draft of the paper. All authors contributed to the article and approved the submitted version.

## FUNDING

GJ‐S and KB‐S receive funding from the University of Washington Center for AIDS Research (UW CFAR P30 AI027757). Research reported in this publication was supported by *Eunice Kennedy Shriver* National Institute of Child Health and Human Development of the National Institutes of Health under grant number UH3HD096906.

## Supporting information




**File S1**: DiSC stepped care placement tool.

**Supplementary Table**: Participant baseline characteristics by Step.

## Data Availability

The data that support the findings of this study will be openly available in the NICHD Data and Specimen Hub (DASH) in 2025. Until available in the DASH repository, the data that support the findings of this study are available from the corresponding author upon reasonable request.
