# Supplementary material for: Data‐informed Stepped Care (DiSC) to improve adolescent and young adult HIV care outcomes in Kenya: a cluster randomized trial
Source: J Int AIDS Soc. 2025 Jul 7;28(Suppl 3):e26501. doi: 10.1002/jia2.26501 (PMC12232485; doi:10.1002/jia2.26501)
Supplement: Supplementary file 1 — File S1: DiSC stepped care placement tool. [file JIA2-28-e26501-s002.docx]

| **Introduction**: At this clinic, we are offering stepped services for adolescents who receive routine care with us. Instead of everyone receiving exactly the same care, we are providing healthcare that is tailored to your needs. We are placing people in different “steps” depending on their healthcare needs. You can also decide to receive the usual care. | | | |
| --- | --- | --- | --- |
|  | | | |
| Date Stepped-care was explained to patient: | Participant UPN: | Age**:** | Would you like to receive “stepped care”? |
| _____________(DD/MM/YYYY) |  |  | - Yes (Proceed to step eligibility screening) - No (Assign to usual care/Step 2) |

|  | **Visit date 1:** | | | | | | | | |  | **Visit date 2:** | | | | | | | | |
| --- | --- | --- | --- | --- | --- | --- | --- | --- | --- | --- | --- | --- | --- | --- | --- | --- | --- | --- | --- |
| **Step 4** | **Viral load >200 copies/ml** | | | - **Yes** | | - **No** | | | - **NA** |  | **Viral load >200 copies/ml** | | | - **Yes** | | - **No** | | | - NA |
|  | *If yes, assign to Step 4*  *If no, proceed to Step 3 questions* | | | | | | | | |  | *If yes, assign to Step 4*  *If no, proceed to Step 3 questions* | | | | | | | | |
|  |  | | | | | | | | |  |  | | | | | | | | |
| **Step 3** | **Missed one or more scheduled visits in last 6 months** | | | | - **Yes** | | | - **No** | |  | **Missed one or more scheduled visits in last 6 months** | | | | - **Yes** | | | - **No** | |
|  | Over the last 2 weeks, how often have you been bothered by the following problems? | | | | | | | | |  | Over the last 2 weeks, how often have you been bothered by the following problems? | | | | | | | | |
|  | Little interest or pleasure in doing things | | | | | | | | |  | Little interest or pleasure in doing things | | | | | | | | |
|  | Not at all (0) | Several days (1) | More than half the days (2) | | | | Nearly every day (3) | | |  | Not at all (0) | Several days (1) | More than half the days (2) | | | | Nearly every day (3) | | |
|  | Feeling down, depressed or hopeless | | | | | | | | |  | Feeling down, depressed or hopeless | | | | | | | | |
|  | Not at all (0) | Several days (1) | More than half the days (2) | | | | Nearly every day (3) | | |  | Not at all (0) | Several days (1) | More than half the days (2) | | | | Nearly every day (3) | | |
|  | **Symptoms of depression (PHQ-2 score ≥3)** | | | | - **Yes** | | | - **No** | |  | **Symptoms of depression (PHQ-2 score ≥3)** | | | | - **Yes** | | | - **No** | |
|  | *If yes to any, assign to Step 3*  *If no to all, proceed to Step 2 questions* | | | | | | | | |  | *If yes to any, assign to Step 3*  *If no to all, proceed to Step 2 questions* | | | | | | | | |
|  |  | | | | | | | | |  |  | | | | | | | | |
| **Step 2** | **Patient requested usual care** | | | | - **Yes** | | | - **No** | |  | **Patient requested usual care** | | | | - **Yes** | | | - **No** | |
|  | **Enrolled within last 6 months** | | | | - **Yes** | | | - **No** | |  | **Enrolled within last 6 months** | | | | - **Yes** | | | - **No** | |
|  | **Opportunistic infection** | | | | - **Yes** | | | - **No** | |  | **Opportunistic infection** | | | | - **Yes** | | | - **No** | |
|  | **Pregnant or breastfeeding** | | | | - **Yes** | | | - **No** | |  | **Pregnant or breastfeeding** | | | | - **Yes** | | | - **No** | |
|  | *If yes to any, assign to Step 2*  *If no to all, proceed to Step 1 questions* | | | | | | | | |  | *If yes to any, assign to Step 2*  *If no to all, proceed to Step 1 questions* | | | | | | | | |
|  |  | | | | | | | | |  |  | | | | | | | | |
| **Step 1** | **On ART for ≥ 12 months** | | | | - **Yes** | | | - **No** | |  | **On ART for ≥ 12 months** | | | | - **Yes** | | | - **No** | |
|  | **No other infection in last 6 months** | | | | - **Yes** | | | - **No** | |  | **No other infection in last 6 months** | | | | - **Yes** | | | - **No** | |
|  | *If yes to all, assign to Step 1*  *If no to any, assign to Step 2* | | | | | | | | |  | *If yes to all, assign to Step 1*  *If no to any, assign to Step 2* | | | | | | | | |
|  |  | | | | | | | | |  |  | | | | | | | | |
| **Step Assigned** | Step assigned today:   - Step 4 - Step 3 - Step 2 - Step 1 | | TCA: (dd/mm/yyyy):  Referred Yes No | | | | | | |  | Step assigned today:   - Step 4 - Step 3 - Step 2 - Step 1 | | TCA: (dd/mm/yyyy):  Referred Yes No | | | | | | |

|  | **Visit date 3:** | | | | | | | | |  | **Visit date 4:** | | | | | | | | |
| --- | --- | --- | --- | --- | --- | --- | --- | --- | --- | --- | --- | --- | --- | --- | --- | --- | --- | --- | --- |
| **Step 4** | **Viral load >200 copies/ml** | | | - **Yes** | | - **No** | | | - **NA** |  | **Viral load >200 copies/ml** | | | - **Yes** | | - **No** | | | - NA |
|  | *If yes, assign to Step 4*  *If no, proceed to Step 3 questions* | | | | | | | | |  | *If yes, assign to Step 4*  *If no, proceed to Step 3 questions* | | | | | | | | |
|  |  | | | | | | | | |  |  | | | | | | | | |
| **Step 3** | **Missed one or more scheduled visits in last 6 months** | | | | - **Yes** | | | - **No** | |  | **Missed one or more scheduled visits in last 6 months** | | | | - **Yes** | | | - **No** | |
|  | Over the last 2 weeks, how often have you been bothered by the following problems? | | | | | | | | |  | Over the last 2 weeks, how often have you been bothered by the following problems? | | | | | | | | |
|  | Little interest or pleasure in doing things | | | | | | | | |  | Little interest or pleasure in doing things | | | | | | | | |
|  | Not at all (0) | Several days (1) | More than half the days (2) | | | | Nearly every day (3) | | |  | Not at all (0) | Several days (1) | More than half the days (2) | | | | Nearly every day (3) | | |
|  | Feeling down, depressed or hopeless | | | | | | | | |  | Feeling down, depressed or hopeless | | | | | | | | |
|  | Not at all (0) | Several days (1) | More than half the days (2) | | | | Nearly every day (3) | | |  | Not at all (0) | Several days (1) | More than half the days (2) | | | | Nearly every day (3) | | |
|  | **Symptoms of depression (PHQ-2 score ≥3)** | | | | - **Yes** | | | - **No** | |  | Symptoms of depression (PHQ-2 score ≥3) | | | | - **Yes** | | | - **No** | |
|  | *If yes to any, assign to Step 3*  *If no to all, proceed to Step 2 questions* | | | | | | | | |  | *If yes to any, assign to Step 3*  *If no to all, proceed to Step 2 questions* | | | | | | | | |
|  |  | | | | | | | | |  |  | | | | | | | | |
| **Step 2** | **Patient requested usual care** | | | | - **Yes** | | | - **No** | |  | **Patient requested usual care** | | | | - **Yes** | | | - **No** | |
|  | **Enrolled within last 6 months** | | | | - **Yes** | | | - **No** | |  | **Enrolled within last 6 months** | | | | - **Yes** | | | - **No** | |
|  | **Opportunistic infection** | | | | - **Yes** | | | - **No** | |  | **Opportunistic infection** | | | | - **Yes** | | | - **No** | |
|  | **Pregnant or breastfeeding** | | | | - **Yes** | | | - **No** | |  | **Pregnant or breastfeeding** | | | | - **Yes** | | | - **No** | |
|  | *If yes to any, assign to Step 2*  *If no to all, proceed to Step 1 questions* | | | | | | | | |  | *If yes to any, assign to Step 2*  *If no to all, proceed to Step 1 questions* | | | | | | | | |
|  |  | | | | | | | | |  |  | | | | | | | | |
| **Step 1** | **On ART for ≥ 12 months** | | | | - **Yes** | | | - **No** | |  | **On ART for ≥ 12 months** | | | | - **Yes** | | | - **No** | |
|  | **No other infection in last 6 months** | | | | - **Yes** | | | - **No** | |  | **No other infection in last 6 months** | | | | - **Yes** | | | - **No** | |
|  | *If yes to all, assign to Step 1*  *If no to any, assign to Step 2* | | | | | | | | |  | *If yes to all, assign to Step 1*  *If no to any, assign to Step 2* | | | | | | | | |
|  |  | | | | | | | | |  |  | | | | | | | | |
| **Step Assigned** | Step assigned today:   - Step 4 - Step 3 - Step 2 - Step 1 | | TCA: (dd/mm/yyyy):  Referred Yes No | | | | | | |  | Step assigned today:   - Step 4 - Step 3 - Step 2 - Step 1 | | TCA: (dd/mm/yyyy):  Referred Yes No | | | | | | |

|  | **Visit date 5:** | | | | | | | | |  | **Visit date 6:** | | | | | | | | |
| --- | --- | --- | --- | --- | --- | --- | --- | --- | --- | --- | --- | --- | --- | --- | --- | --- | --- | --- | --- |
| **Step 4** | **Viral load >200 copies/ml** | | | - **Yes** | | - **No** | | | - **NA** |  | **Viral load >200 copies/ml** | | | - **Yes** | | - **No** | | | - NA |
|  | *If yes, assign to Step 4*  *If no, proceed to Step 3 questions* | | | | | | | | |  | *If yes, assign to Step 4*  *If no, proceed to Step 3 questions* | | | | | | | | |
|  |  | | | | | | | | |  |  | | | | | | | | |
| **Step 3** | **Missed one or more scheduled visits in last 6 months** | | | | - **Yes** | | | - **No** | |  | **Missed one or more scheduled visits in last 6 months** | | | | - **Yes** | | | - **No** | |
|  | Over the last 2 weeks, how often have you been bothered by the following problems? | | | | | | | | |  | Over the last 2 weeks, how often have you been bothered by the following problems? | | | | | | | | |
|  | Little interest or pleasure in doing things | | | | | | | | |  | Little interest or pleasure in doing things | | | | | | | | |
|  | Not at all (0) | Several days (1) | More than half the days (2) | | | | Nearly every day (3) | | |  | Not at all (0) | Several days (1) | More than half the days (2) | | | | Nearly every day (3) | | |
|  | Feeling down, depressed or hopeless | | | | | | | | |  | Feeling down, depressed or hopeless | | | | | | | | |
|  | Not at all (0) | Several days (1) | More than half the days (2) | | | | Nearly every day (3) | | |  | Not at all (0) | Several days (1) | More than half the days (2) | | | | Nearly every day (3) | | |
|  | **Symptoms of depression (PHQ-2 score ≥3)** | | | | - **Yes** | | | - **No** | |  | Symptoms of depression (PHQ-2 score ≥3) | | | | - **Yes** | | | - **No** | |
|  | *If yes to any, assign to Step 3*  *If no to all, proceed to Step 2 questions* | | | | | | | | |  | *If yes to any, assign to Step 3*  *If no to all, proceed to Step 2 questions* | | | | | | | | |
|  |  | | | | | | | | |  |  | | | | | | | | |
| **Step 2** | **Patient requested usual care** | | | | - **Yes** | | | - **No** | |  | **Patient requested usual care** | | | | - **Yes** | | | - **No** | |
|  | **Enrolled within last 6 months** | | | | - **Yes** | | | - **No** | |  | **Enrolled within last 6 months** | | | | - **Yes** | | | - **No** | |
|  | **Opportunistic infection** | | | | - **Yes** | | | - **No** | |  | **Opportunistic infection** | | | | - **Yes** | | | - **No** | |
|  | **Pregnant or breastfeeding** | | | | - **Yes** | | | - **No** | |  | **Pregnant or breastfeeding** | | | | - **Yes** | | | - **No** | |
|  | *If yes to any, assign to Step 2*  *If no to all, proceed to Step 1 questions* | | | | | | | | |  | *If yes to any, assign to Step 2*  *If no to all, proceed to Step 1 questions* | | | | | | | | |
|  |  | | | | | | | | |  |  | | | | | | | | |
| **Step 1** | **On ART for ≥ 12 months** | | | | - **Yes** | | | - **No** | |  | **On ART for ≥ 12 months** | | | | - **Yes** | | | - **No** | |
|  | **No other infection in last 6 months** | | | | - **Yes** | | | - **No** | |  | **No other infection in last 6 months** | | | | - **Yes** | | | - **No** | |
|  | *If yes to all, assign to Step 1*  *If no to any, assign to Step 2* | | | | | | | | |  | *If yes to all, assign to Step 1*  *If no to any, assign to Step 2* | | | | | | | | |
|  |  | | | | | | | | |  |  | | | | | | | | |
| **Step Assigned** | Step assigned today:   - Step 4 - Step 3 - Step 2 - Step 1 | | TCA: (dd/mm/yyyy):  Referred Yes No | | | | | | |  | Step assigned today:   - Step 4 - Step 3 - Step 2 - Step 1 | | TCA: (dd/mm/yyyy):  Referred Yes No | | | | | | |

|  | **Visit date 7:** | | | | | | | | |  | **Visit date 8:** | | | | | | | | |
| --- | --- | --- | --- | --- | --- | --- | --- | --- | --- | --- | --- | --- | --- | --- | --- | --- | --- | --- | --- |
| **Step 4** | **Viral load >200 copies/ml** | | | - **Yes** | | - **No** | | | - **NA** |  | **Viral load >200 copies/ml** | | | - **Yes** | | - **No** | | | - NA |
|  | *If yes, assign to Step 4*  *If no, proceed to Step 3 questions* | | | | | | | | |  | *If yes, assign to Step 4*  *If no, proceed to Step 3 questions* | | | | | | | | |
|  |  | | | | | | | | |  |  | | | | | | | | |
| **Step 3** | **Missed one or more scheduled visits in last 6 months** | | | | - **Yes** | | | - **No** | |  | **Missed one or more scheduled visits in last 6 months** | | | | - **Yes** | | | - **No** | |
|  | Over the last 2 weeks, how often have you been bothered by the following problems? | | | | | | | | |  | Over the last 2 weeks, how often have you been bothered by the following problems? | | | | | | | | |
|  | Little interest or pleasure in doing things | | | | | | | | |  | Little interest or pleasure in doing things | | | | | | | | |
|  | Not at all (0) | Several days (1) | More than half the days (2) | | | | Nearly every day (3) | | |  | Not at all (0) | Several days (1) | More than half the days (2) | | | | Nearly every day (3) | | |
|  | Feeling down, depressed or hopeless | | | | | | | | |  | Feeling down, depressed or hopeless | | | | | | | | |
|  | Not at all (0) | Several days (1) | More than half the days (2) | | | | Nearly every day (3) | | |  | Not at all (0) | Several days (1) | More than half the days (2) | | | | Nearly every day (3) | | |
|  | **Symptoms of depression (PHQ-2 score ≥3)** | | | | - **Yes** | | | - **No** | |  | Symptoms of depression (PHQ-2 score ≥3) | | | | - **Yes** | | | - **No** | |
|  | *If yes to any, assign to Step 3*  *If no to all, proceed to Step 2 questions* | | | | | | | | |  | *If yes to any, assign to Step 3*  *If no to all, proceed to Step 2 questions* | | | | | | | | |
|  |  | | | | | | | | |  |  | | | | | | | | |
| **Step 2** | **Patient requested usual care** | | | | - **Yes** | | | - **No** | |  | **Patient requested usual care** | | | | - **Yes** | | | - **No** | |
|  | **Enrolled within last 6 months** | | | | - **Yes** | | | - **No** | |  | **Enrolled within last 6 months** | | | | - **Yes** | | | - **No** | |
|  | **Opportunistic infection** | | | | - **Yes** | | | - **No** | |  | **Opportunistic infection** | | | | - **Yes** | | | - **No** | |
|  | **Pregnant or breastfeeding** | | | | - **Yes** | | | - **No** | |  | **Pregnant or breastfeeding** | | | | - **Yes** | | | - **No** | |
|  | *If yes to any, assign to Step 2*  *If no to all, proceed to Step 1 questions* | | | | | | | | |  | *If yes to any, assign to Step 2*  *If no to all, proceed to Step 1 questions* | | | | | | | | |
|  |  | | | | | | | | |  |  | | | | | | | | |
| **Step 1** | **On ART for ≥ 12 months** | | | | - **Yes** | | | - **No** | |  | **On ART for ≥ 12 months** | | | | - **Yes** | | | - **No** | |
|  | **No other infection in last 6 months** | | | | - **Yes** | | | - **No** | |  | **No other infection in last 6 months** | | | | - **Yes** | | | - **No** | |
|  | *If yes to all, assign to Step 1*  *If no to any, assign to Step 2* | | | | | | | | |  | *If yes to all, assign to Step 1*  *If no to any, assign to Step 2* | | | | | | | | |
|  |  | | | | | | | | |  |  | | | | | | | | |
| **Step Assigned** | Step assigned today:   - Step 4 - Step 3 - Step 2 - Step 1 | | TCA: (dd/mm/yyyy):  Referred Yes No | | | | | | |  | Step assigned today:   - Step 4 - Step 3 - Step 2 - Step 1 | | TCA: (dd/mm/yyyy):  Referred Yes No | | | | | | |

|  | **Visit date 9:** | | | | | | | | |  | **Visit date 10:** | | | | | | | | |
| --- | --- | --- | --- | --- | --- | --- | --- | --- | --- | --- | --- | --- | --- | --- | --- | --- | --- | --- | --- |
| **Step 4** | **Viral load >200 copies/ml** | | | - **Yes** | | - **No** | | | - **NA** |  | **Viral load >200 copies/ml** | | | - **Yes** | | - **No** | | | - NA |
|  | *If yes, assign to Step 4*  *If no, proceed to Step 3 questions* | | | | | | | | |  | *If yes, assign to Step 4*  *If no, proceed to Step 3 questions* | | | | | | | | |
|  |  | | | | | | | | |  |  | | | | | | | | |
| **Step 3** | **Missed one or more scheduled visits in last 6 months** | | | | - **Yes** | | | - **No** | |  | **Missed one or more scheduled visits in last 6 months** | | | | - **Yes** | | | - **No** | |
|  | Over the last 2 weeks, how often have you been bothered by the following problems? | | | | | | | | |  | Over the last 2 weeks, how often have you been bothered by the following problems? | | | | | | | | |
|  | Little interest or pleasure in doing things | | | | | | | | |  | Little interest or pleasure in doing things | | | | | | | | |
|  | Not at all (0) | Several days (1) | More than half the days (2) | | | | Nearly every day (3) | | |  | Not at all (0) | Several days (1) | More than half the days (2) | | | | Nearly every day (3) | | |
|  | Feeling down, depressed or hopeless | | | | | | | | |  | Feeling down, depressed or hopeless | | | | | | | | |
|  | Not at all (0) | Several days (1) | More than half the days (2) | | | | Nearly every day (3) | | |  | Not at all (0) | Several days (1) | More than half the days (2) | | | | Nearly every day (3) | | |
|  | **Symptoms of depression (PHQ-2 score ≥3)** | | | | - **Yes** | | | - **No** | |  | Symptoms of depression (PHQ-2 score ≥3) | | | | - **Yes** | | | - **No** | |
|  | *If yes to any, assign to Step 3*  *If no to all, proceed to Step 2 questions* | | | | | | | | |  | *If yes to any, assign to Step 3*  *If no to all, proceed to Step 2 questions* | | | | | | | | |
|  |  | | | | | | | | |  |  | | | | | | | | |
| **Step 2** | **Patient requested usual care** | | | | - **Yes** | | | - **No** | |  | **Patient requested usual care** | | | | - **Yes** | | | - **No** | |
|  | **Enrolled within last 6 months** | | | | - **Yes** | | | - **No** | |  | **Enrolled within last 6 months** | | | | - **Yes** | | | - **No** | |
|  | **Opportunistic infection** | | | | - **Yes** | | | - **No** | |  | **Opportunistic infection** | | | | - **Yes** | | | - **No** | |
|  | **Pregnant or breastfeeding** | | | | - **Yes** | | | - **No** | |  | **Pregnant or breastfeeding** | | | | - **Yes** | | | - **No** | |
|  | *If yes to any, assign to Step 2*  *If no to all, proceed to Step 1 questions* | | | | | | | | |  | *If yes to any, assign to Step 2*  *If no to all, proceed to Step 1 questions* | | | | | | | | |
|  |  | | | | | | | | |  |  | | | | | | | | |
| **Step 1** | **On ART for ≥ 12 months** | | | | - **Yes** | | | - **No** | |  | **On ART for ≥ 12 months** | | | | - **Yes** | | | - **No** | |
|  | **No other infection in last 6 months** | | | | - **Yes** | | | - **No** | |  | **No other infection in last 6 months** | | | | - **Yes** | | | - **No** | |
|  | *If yes to all, assign to Step 1*  *If no to any, assign to Step 2* | | | | | | | | |  | *If yes to all, assign to Step 1*  *If no to any, assign to Step 2* | | | | | | | | |
|  |  | | | | | | | | |  |  | | | | | | | | |
| **Step Assigned** | Step assigned today:   - Step 4 - Step 3 - Step 2 - Step 1 | | TCA: (dd/mm/yyyy):  Referred Yes No | | | | | | |  | Step assigned today:   - Step 4 - Step 3 - Step 2 - Step 1 | | TCA: (dd/mm/yyyy):  Referred Yes No | | | | | | |

|  | **Visit date 11:** | | | | | | | | |  | **Visit date 12:** | | | | | | | | |
| --- | --- | --- | --- | --- | --- | --- | --- | --- | --- | --- | --- | --- | --- | --- | --- | --- | --- | --- | --- |
| **Step 4** | **Viral load >200 copies/ml** | | | - **Yes** | | - **No** | | | - **NA** |  | **Viral load >200 copies/ml** | | | - **Yes** | | - **No** | | | - NA |
|  | *If yes, assign to Step 4*  *If no, proceed to Step 3 questions* | | | | | | | | |  | *If yes, assign to Step 4*  *If no, proceed to Step 3 questions* | | | | | | | | |
|  |  | | | | | | | | |  |  | | | | | | | | |
| **Step 3** | **Missed one or more scheduled visits in last 6 months** | | | | - **Yes** | | | - **No** | |  | **Missed one or more scheduled visits in last 6 months** | | | | - **Yes** | | | - **No** | |
|  | Over the last 2 weeks, how often have you been bothered by the following problems? | | | | | | | | |  | Over the last 2 weeks, how often have you been bothered by the following problems? | | | | | | | | |
|  | Little interest or pleasure in doing things | | | | | | | | |  | Little interest or pleasure in doing things | | | | | | | | |
|  | Not at all (0) | Several days (1) | More than half the days (2) | | | | Nearly every day (3) | | |  | Not at all (0) | Several days (1) | More than half the days (2) | | | | Nearly every day (3) | | |
|  | Feeling down, depressed or hopeless | | | | | | | | |  | Feeling down, depressed or hopeless | | | | | | | | |
|  | Not at all (0) | Several days (1) | More than half the days (2) | | | | Nearly every day (3) | | |  | Not at all (0) | Several days (1) | More than half the days (2) | | | | Nearly every day (3) | | |
|  | **Symptoms of depression (PHQ-2 score ≥3)** | | | | - **Yes** | | | - **No** | |  | Symptoms of depression (PHQ-2 score ≥3) | | | | - **Yes** | | | - **No** | |
|  | *If yes to any, assign to Step 3*  *If no to all, proceed to Step 2 questions* | | | | | | | | |  | *If yes to any, assign to Step 3*  *If no to all, proceed to Step 2 questions* | | | | | | | | |
|  |  | | | | | | | | |  |  | | | | | | | | |
| **Step 2** | **Patient requested usual care** | | | | - **Yes** | | | - **No** | |  | **Patient requested usual care** | | | | - **Yes** | | | - **No** | |
|  | **Enrolled within last 6 months** | | | | - **Yes** | | | - **No** | |  | **Enrolled within last 6 months** | | | | - **Yes** | | | - **No** | |
|  | **Opportunistic infection** | | | | - **Yes** | | | - **No** | |  | **Opportunistic infection** | | | | - **Yes** | | | - **No** | |
|  | **Pregnant or breastfeeding** | | | | - **Yes** | | | - **No** | |  | **Pregnant or breastfeeding** | | | | - **Yes** | | | - **No** | |
|  | *If yes to any, assign to Step 2*  *If no to all, proceed to Step 1 questions* | | | | | | | | |  | *If yes to any, assign to Step 2*  *If no to all, proceed to Step 1 questions* | | | | | | | | |
|  |  | | | | | | | | |  |  | | | | | | | | |
| **Step 1** | **On ART for ≥ 12 months** | | | | - **Yes** | | | - **No** | |  | **On ART for ≥ 12 months** | | | | - **Yes** | | | - **No** | |
|  | **No other infection in last 6 months** | | | | - **Yes** | | | - **No** | |  | **No other infection in last 6 months** | | | | - **Yes** | | | - **No** | |
|  | *If yes to all, assign to Step 1*  *If no to any, assign to Step 2* | | | | | | | | |  | *If yes to all, assign to Step 1*  *If no to any, assign to Step 2* | | | | | | | | |
|  |  | | | | | | | | |  |  | | | | | | | | |
| **Step Assigned** | Step assigned today:   - Step 4 - Step 3 - Step 2 - Step 1 | | TCA: (dd/mm/yyyy):  Referred Yes No | | | | | | |  | Step assigned today:   - Step 4 - Step 3 - Step 2 - Step 1 | | TCA: (dd/mm/yyyy):  Referred Yes No | | | | | | |
