# Supplementary material for: Data‐informed Stepped Care (DiSC) to improve adolescent and young adult HIV care outcomes in Kenya: a cluster randomized trial
Source: J Int AIDS Soc. 2025 Jul 7;28(Suppl 3):e26501. doi: 10.1002/jia2.26501 (PMC12232485; doi:10.1002/jia2.26501)
Supplement: Supplementary file 2 — Supplementary Table: Participant baseline characteristics by Step. [file JIA2-28-e26501-s001.docx]

**Supplementary Table. Participant baseline characteristics by Step**

| **Characteristic** | Control (N=1,016)  *n (%) or median (IQR)* | Step 1 (N=571)  *n (%) or median (IQR)* | Step 2 (N=118)  *n (%) or median (IQR)* | Step 3 (N=100)  *n (%) or median (IQR)* | Step 4 (N=92)  *n (%) or median (IQR)* |
| --- | --- | --- | --- | --- | --- |
| Age (years) | 17.0 (15.0, 19.0) | 16.0 (13.0, 18.0) | 20.0 (16.0, 22.0) | 18.0 (15.0, 21.0) | 15.0 (13.0, 18.0) |
| Gender |  |  |  |  |  |
| Male | 443 (43.6%) | 251 (44.0%) | 17 (14.4%) | 40 (40.0%) | 44 (47.8%) |
| Female | 570 (56.2%) | 318 (55.8%) | 101 (85.6%) | 60 (60.0%) | 47 (51.1%) |
| Currently in school | 806 (79.6%) | 508 (89.0%) | 50 (42.4%) | 67 (67.0%) | 77 (83.7%) |
| At least one parent alive | 854 (84.1%) | 480 (84.1%) | 94 (79.7%) | 83 (83.8%) | 70 (76.1%) |
| At least one parent died | 570 (56.1%) | 310 (54.3%) | 72 (61.0%) | 58 (58.6%) | 58 (63.0%) |
| Depression ^a^ |  |  |  |  |  |
| None/minimal (0-4) | 906 (91.3%) | 520 (94.7%) | 109 (96.5%) | 74 (74.7%) | 80 (89.9%) |
| At least mild (5+) | 86 (8.7%) | 29 (5.3%) | 4 (3.5%) | 25 (25.3%) | 9 (10.1%) |
| Anxiety ^b^ |  |  |  |  |  |
| None/minimal (0-4) | 967 (96.9%) | 539 (96.8%) | 113 (95.8%) | 86 (88.7%) | 86 (95.6%) |
| At least mild (5+) | 31 (3.1%) | 18 (3.2%) | 5 (4.2%) | 11 (11.3%) | 4 (4.4%) |
| Any alcohol or drug use in life ^c^ | 79 (7.8%) | 38 (6.7%) | 15 (12.7%) | 10 (10.0%) | 4 (4.3%) |
| Social support ^d^ | 924 (96.6%) | 502 (95.3%) | 103 (94.5%) | 84 (89.4%) | 82 (96.5%) |
| Resilience ^e^ | 930 (93.2%) | 515 (92.5%) | 103 (89.6%) | 87 (89.7%) | 88 (95.7%) |
| Adherence self-efficacy ^f^ | 979 (98.9%) | 549 (98.2%) | 116 (98.3%) | 98 (98.0%) | 87 (100.0%) |
| Stigma ^g^ | 351 (37.1%) | 162 (31.2%) | 44 (40.0%) | 37 (43.5%) | 23 (27.4%) |
| Part of a peer support group | 499 (49.7%) | 281 (50.3%) | 30 (25.4%) | 43 (43.0%) | 48 (52.7%) |
| Violence in the last 6 months | 54 (5.3%) | 39 (6.8%) | 11 (9.3%) | 8 (8.0%) | 4 (4.3%) |
| Transport to clinic ≥1 hour | 479 (47.9%) | 232 (42.0%) | 54 (45.8%) | 35 (35.7%) | 28 (30.4%) |
| Wait time in clinic ≥1 hour | 97 (9.6%) | 46 (8.1%) | 6 (5.2%) | 3 (3.0%) | 3 (3.3%) |
| Use at least 1 differentiated service delivery (DSD) model | 334 (33.0%) | 202 (35.6%) | 41 (34.7%) | 45 (45.5%) | 42 (45.7%) |
| Report service needed but not received | 580 (57.1%) | 323 (56.6%) | 86 (72.9%) | 66 (66.0%) | 43 (46.7%) |
| Able to come for appointments without help from caregiver | 794 (84.3%) | 429 (76.5%) | 100 (89.3%) | 84 (85.7%) | 70 (77.8%) |
| Able to take medication without reminders from caregiver | 892 (94.9%) | 499 (89.4%) | 108 (98.2%) | 90 (91.8%) | 77 (85.6%) |
| Always coming to this clinic by yourself | 694 (68.6%) | 321 (56.4%) | 94 (79.7%) | 64 (64.0%) | 57 (62.0%) |
| Other person in charge of healthcare decision | 759 (75.0%) | 462 (81.3%) | 70 (59.3%) | 70 (70.0%) | 77 (84.6%) |
| Know HIV status of last partner | 258 (75.2%) | 82 (73.2%) | 54 (73.0%) | 28 (70.0%) | 13 (92.9%) |
| Disclosed to anyone | 995 (97.9%) | 560 (98.1%) | 116 (98.3%) | 100 (100.0%) | 91 (98.9%) |
| Antiretroviral therapy regimen |  |  |  |  |  |
| Dolutegravir (DTG)-based | 348 (35.4%) | 211 (38.1%) | 81 (69.8%) | 52 (52.0%) | 15 (16.3%) |
| Efavirenz (EFV)-based | 222 (22.6%) | 122 (22.0%) | 9 (7.8%) | 19 (19.0%) | 27 (29.3%) |
| Lopinavir/ritonavir (LPV/r)-based | 89 (9.1%) | 38 (6.9%) | 3 (2.6%) | 5 (5.0%) | 14 (15.2%) |
| Nevirapine (NVP)-based | 281 (28.6%) | 168 (30.3%) | 18 (15.5%) | 22 (22.0%) | 32 (34.8%) |
| Other | 43 (4.4%) | 15 (2.7%) | 5 (4.3%) | 2 (2.0%) | 4 (4.3%) |

^a^ evaluated using Patient Health Questionnaire-9 (PHQ-9) with a score of 0-4 indicating none/minimal depression, 5-9 mild depression, 10-14 moderate depression, ≥15 severe depression; ^b^ evaluated using General Anxiety Disorder-7 (GAD-7) with a score of 0-4 indicating none/minimal anxiety, 5-9 mild anxiety, 10-14 moderate anxiety, ≥15 severe anxiety; ^c^ using WHO Alcohol, Smoking and Substance Involvement Screening Test for Young people (ASSIST-Y); ^d^ evaluated using Multidimensional Scale of Perceived Social Support (MSPSS) 12-item scale with a score >3 indicating high social support; ^e^ evaluated using Connor-Davidson Resilience Scale-2 (CDRS-2) with a score ≥3 indicating high resilience; ^f^ evaluated using Adherence Self-Efficacy Scale (ASES) with a score ≥5 indicating high adherence; ^g^ evaluated using Youth HIV Brief Stigma Scale (YHBSS) with a score ≥3 indicating high stigma experience
